# Predictive Value of High ICAM-1 Level for Poor Treatment Response to Low-Dose Decitabine in Adult Corticosteroid Resistant ITP Patients

**DOI:** 10.3389/fimmu.2021.689663

**Published:** 2021-07-13

**Authors:** Chaoyang Li, Lizhen Li, Meng Sun, Jianzhi Sun, Linlin Shao, Miao Xu, Yu Hou, Jun Peng, Lin Wang, Ming Hou

**Affiliations:** ^1^ Department of Hematology, Qilu Hospital, Cheeloo College of Medicine, Shandong University, Jinan, China; ^2^ Jinan Vocational College of Nursing, Jinan, China; ^3^ Shandong Provincial Key Laboratory of Immunohematology, Qilu Hospital, Cheeloo College of Medicine, Shandong University, Jinan, China; ^4^ Leading Research Group of Scientific Innovation, Department of Science and Technology of Shandong Province, Qilu Hospital, Cheeloo College of Medicine, Shandong University, Jinan, China

**Keywords:** ICAM-1, immune thrombocytopenia, decitabine, endothelia cell dysfunction, predict

## Abstract

Primary immune thrombocytopenia (ITP) is an autoimmune hemorrhagic disease. Endothelial cell activation/injury has been found in some autoimmune diseases including SLE, systemic sclerosis, and rheumatoid arthritis, but its role in ITP pathogenesis remains unclear. This study attempted to elucidate the correlation between endothelial dysfunction and disease severity of ITP and find related markers to predict response to low-dose decitabine treatment. Compared with healthy volunteers, higher plasma levels of soluble intercellular adhesion molecule-1 (ICAM-1), vascular endothelial growth factor (VEGF), and Angiopoietin-2 were found in adult corticosteroid resistant ITP patients. Notably, ICAM-1 levels were negatively correlated with the platelet count, and positively associated with the bleeding score. Recently, we have reported the efficacy and safety of low-dose decitabine in adult patients with ITP who failed for the first line therapies. Here, we evaluated the correlation of plasma ICAM-1 level with the efficacy of low-dose decitabine therapy for corticosteroid resistant ITP. A total of 29 adult corticosteroid resistant ITP patients who received consecutive treatments of low-dose decitabine were enrolled in this study. Fourteen patients showed response (nine showed complete response and five showed partial response). The levels of ICAM-1 before and after treatment were significantly higher in the non-responsive ITP patients than in the responsive patients. As shown in the multivariable logistic regression model, the odds of developing no-response to low-dose decitabine increased by 36.8% for per 5 ng/ml increase in plasma ICAM-1 level [odds ratio (OR) 1.368, 95% confidence interval (CI): 1.060 to 1.764]. In summary, this was the first study to elucidate the relationship between endothelial dysfunction and corticosteroid resistant ITP and identify the potential predictive value of ICAM-1 level for response to low-dose decitabine.

## Introduction

Primary immune thrombocytopenia (ITP) is the most common hemorrhagic disorder, characterized by decreased platelet number and elevated bleeding risk (1, 2). Increased platelet destruction and impaired platelet production induced by immune intolerance are mainly involved in the pathogenesis of ITP (3–5). There has been rapid development of novel treatments for ITP in the last two decades, such as thrombopoietin receptor agonists, rituximab, and fostamatinib (6–9). Recently, low-dose decitabine has been proven to be efficient and safe in refractory ITP patients in a prospective and multicenter study (10) and therefore has become a vital therapeutic option for ITP patients in China (11). However, numerous ITP patients failed to respond or relapsed after these treatments (12). Therefore, further research is necessary to clarify the other underlying pathogenesis of ITP.

Endothelium forms a vital and selective membrane barrier, which could regulate the transportation of water, solutes, cytokines and blood components in the tissue compartment in healthy or disease conditions (13). Previous reports have shown that increased endothelial cell activation and disruption of the vascular integrity correlates with thrombocytopenia in critically ill patients (14). Increasing evidence has indicated that endothelial dysfunction contributes to recruitment of leukocytes and activation of platelets by expressing adhesion molecules and releasing cytokines, including tumor necrosis factor-alpha (TNF-α), interferon gamma (IFN-*γ*), and interleukin-1 (IL-1), which lead to development of inflammation (15–17). Chronic inflammation is involved in the perturbation of immune tolerance in autoimmune diseases (18). Although endothelial dysfunction has been shown to participate in some autoimmune diseases including SLE, systemic sclerosis, and rheumatoid arthritis (19–21), whether it is involved in ITP pathogenesis remains unclear and its potential prognostic/predictive role requires further investigation.

In the current study, we aimed to identify soluble plasma markers of endothelial dysfunction in healthy donors and adult corticosteroid resistant ITP patients. The association between endothelial dysfunction and disease severity represented by platelet counts and bleeding risk was evaluated in this study. Although low-dose decitabine has been a vital therapeutic option for corticosteroid resistant ITP patients in China, it remains unclear how to identify patients who probably benefit from this therapy. We measured plasma intercellular adhesion molecule-1 (ICAM-1) and Angiopoietin-2 levels in 29 corticosteroid resistant ITP patients during their consecutive treatments of low-dose decitabine and assessed the predictive value of the two markers for treatment response.

## Methods

### Study Design and Patient Identification

First, we analyzed plasma levels of endothelial cell activation markers in adult corticosteroid resistant ITP patients and healthy volunteers to determine the association between endothelial cell activation and disease severity. Eighty eight adult corticosteroid resistant ITP patients were randomly enrolled between July 2017 and January 2020 who did not undergo any ITP-specific therapy for at least one month. All patients were recruited at the Department of Hematology, Qilu Hospital, Cheeloo College of Medicine, Shandong University, Jinan, China. ITP diagnosis was based on the previously established criteria (22). All the enrolled participants were aged ≥18 years, with a platelet count <30 × 10^9^/L. Previous ITP specific treatments mainly included corticosteroids, intravenous immunoglobulin, thrombopoietin-receptor agonists (rhTPO, eltrombopag), and rituximab (**Table 1**).

**Table 1:** Demographic and characteristics.

	Normal (n = 30)	ITP (n = 88)
**Age (years), median (range)**	32 (19–56)	48 (15–84)
**Female, % (n)**	53 (16)	66 (58)
**Platelet count (10^9^/L),** **median (range)**	246 (195–285)	10 (1–83)
**Bleeding score, % (n)**		
0		20 (18)
1		42 (37)
2		16 (14)
3		14 (12)
4		3 (3)
5		2 (2)
6		2 (2)
**Previous therapies, % (n)**		
Corticosteroid		100 (88)
rhTPO		39 (34)
Eltrombopag		36 (32)
Rituximab		45 (40)
Intravenous Immunoglobulin		70 (62)

Next, we followed up 29 adult corticosteroid resistant ITP patients who received consecutive low-dose decitabine therapy until achieving response or 90 days from the time of enrollment, whichever occurred first. Decitabine was administered at 3.5 mg/m^2^ intravenously for three consecutive days per cycle, for three cycles, with a four-week interval between cycles (10). Plasma samples were collected at enrollment and on response day in responders or 90th days in non-responders to detect endothelial cell activation marker levels. Cases complicated with diabetes, hypertension, cardiovascular diseases, pregnancy, and active infection were excluded. Thirty healthy volunteers were recruited as controls. The study was approved by the Medical Ethical Committee of Qilu Hospital, Shandong University. Informed consent was obtained from all patients before enrollment in accordance with the Declaration of Helsinki.

### Data Collection

The medical data were collected from the medical therapy records. Bleeding scores were calculated at the time of admission, according to bleeding rating system in ITP (23). This bleeding rating system is calculated by the sum of age scale and bleeding manifestation scale (the highest score among all the bleeding scores). The bleeding rating system was preliminarily evaluated in ITP patients, and bleeding scale was in excellent agreement with ITP-specific bleeding assessment tool (ITP-BAT), while it was less time-consuming than ITP-BAT (3 *vs*. 7 min, p < 0.001). Laboratory values were recorded from the latest data of the enrolled sample collection.

### Plasma Protein Analysis

EDTA anti-coagulated plasma samples were stored at −80°C after peripheral blood samples were drawn. Angiopoietin-1, Angiopoietin-2, ICAM-1, and VEGF were measured by ELISA using SYNERGY H1 microplate reader (BioTek, Winooski, VT, USA) with four-fold diluted plasma samples, according to protocol of the manufacturer (R&D Systems, Minneapolis, MN, USA). Soluble E-selectin and fractalkine were measured by BD cytometric bead array (BD Biosciences, San Diego, CA, USA) using GALLIOS flow cytometer (Beckman Coulter, Brea, CA, USA).

### Statistical Analysis

Statistical analyses were conducted using SPSS 17.0 (SPSS Software, USA) and GraphPad Prism 7 (GraphPad Software Inc, USA). Continuous non-parametric variables were analyzed using the Mann–Whitney U test. Continuous parametric variables were analyzed using the Student’s t-test. Categorical data were analyzed using chi-square test or Fisher’s exact test. For the candidate association between relevant clinical characteristics, all variables were log transformed and Pearson’s correlation coefficient analyses were conducted. Univariate logistic regression models and multivariate logistic regression models were built to determine the clinical characteristics that were predictors for therapeutic response. Two-tailed p-values <0.05 were considered statistically significant.

## Results

### Demographic and Clinical Characteristics

A total of 88 corticosteroid resistant ITP patients were enrolled (66% female, median age 48 years, median platelet count 10 × 10^9^/L) (**Table 1**). Bleeding scores were assessed at the day of enrollment. The percentage of patients graded as score 0, 1, 2, 3, 4, 5, and 6 was 20, 42, 16, 14, 3, 2, and 2% respectively (**Table 1**).

### Angiopoietin-2, ICAM-1, and VEGF Concentrations Were Increased in Corticosteroid Resistant ITP Patients

**Figure 1:**
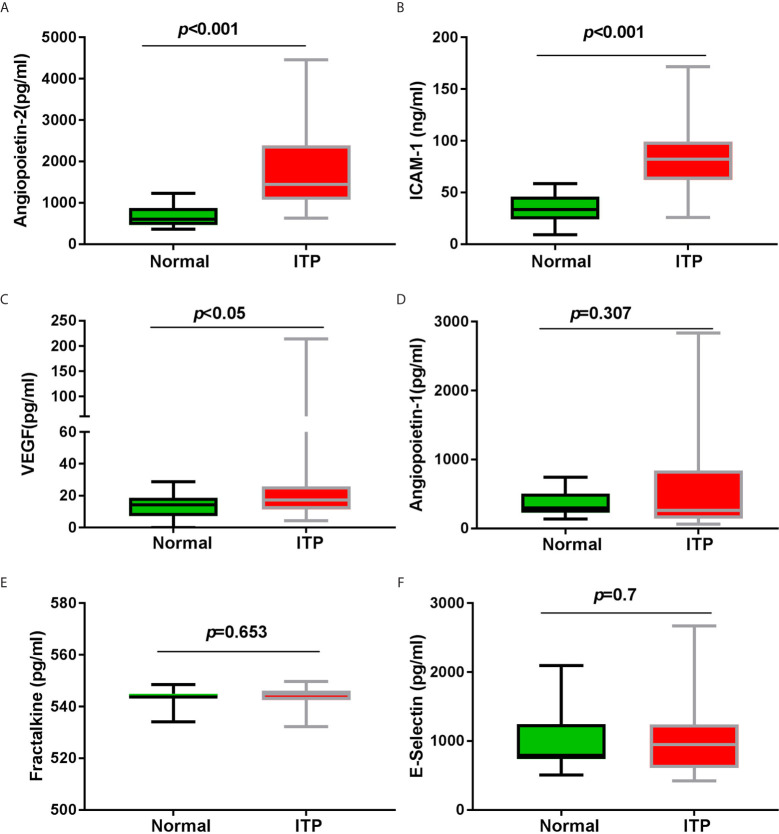
The biomarkers of endothelial activation and vascular integrity. **(A)** Plasma soluble Angiopoietin-2 concentration, **(B)** plasma soluble ICAM-1 concentration, and **(C)** plasma soluble VEGF concentration were significantly higher in adult corticosteroid resistant ITP patients (n = 88 compared with healthy volunteers n = 30). However, **(D)** plasma soluble Angiopoietin-1 concentration, **(E)** plasma soluble Fractalkine concentration, and **(F)** plasma soluble E-selectin concentration between adult corticosteroid resistant ITP patients and healthy volunteers show no markedly difference. Significance between these two groups was determined by Student’s t-test. Data are expressed as box-whisker plots: the central horizontal line, lower and upper of boxes depict the median, first quartile, and third quartile respectively; and the whisker extends to the minimal and maximal data point.

Plasma soluble Angiopoietin-2 concentrations were markedly elevated in corticosteroid resistant ITP patients [median 1,446 (IQR 1,079, 2,391) pg/ml] compared with healthy volunteers [606 (464, 872) pg/ml; p < 0.001] (**Figure 1A**). Additionally, plasma soluble ICAM-1 and VEGF levels were significantly increased in these patients [82 (62, 99) ng/ml; 17 (12, 26) pg/ml] compared with healthy volunteers [33 (24, 46) ng/ml, p < 0.001; 14 (7, 19) pg/ml, p <0.05] (**Figures 1B, C**). Pearson’s correlation coefficient analysis showed no correlation between Angiopoietin-2, ICAM-1, and VEGF (data not shown).

As the antagonist of Angiopoietin-2, Angiopoietin-1 concentrations between adult corticosteroid resistant ITP patients and healthy volunteers did not show significant difference (**Figure 1D**). There was no significant difference in the other two vascular endothelium activation markers, Fractalkine and E-selectin, between these patients and healthy volunteers (**Figures 1E, F**).

### Plasma ICAM-1 Levels Were Correlated With Platelet Count and Bleeding Severity

**Figure 2:**
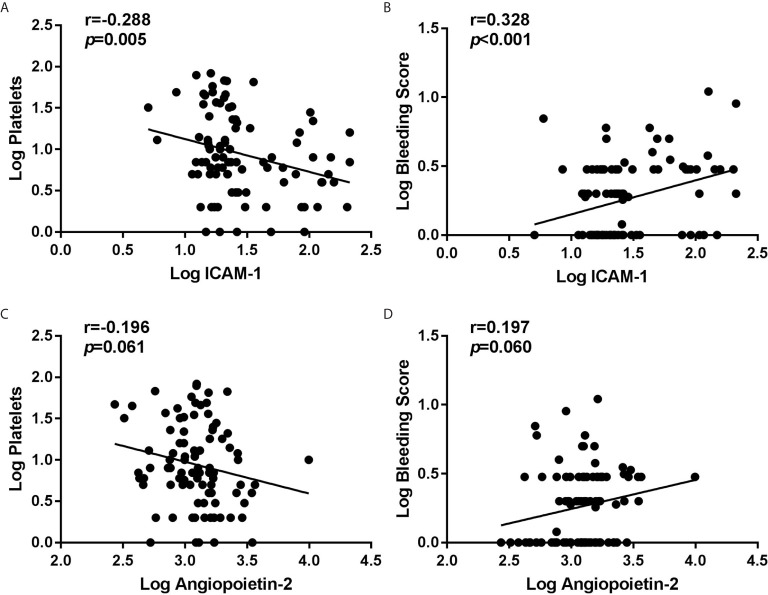
Correlation between initial plasma Angiopoietin-2/ICAM-1 and clinical variables in 88 corticosteroid resistant ITP patients. By Pearson’s correlation coefficient analyses, we found negative correlation between ICAM-1 and platelet count **(A)**, but no obvious association between Angiopoietin-2 and platelet count **(C)**. Meanwhile, we found positive correlation between ICAM-1 and bleeding score **(B)**, but no significant association between Angiopoietin-2 and bleeding score **(D)**. All variables are log transformed.

The associations of Angiopoietin-2 and ICAM-1 concentrations with clinical characteristics and laboratory parameters were analyzed. The results showed that ICAM-1 were significantly negatively associated with the platelet count (**Figure 2A**), but positively associated with bleeding scores (**Figure 2B**). But we found that Angiopoietin-2 was neither associated with platelet count nor bleeding score (**Figures 2C, D**).

In the subgroup analysis stratified by baseline platelet count, the soluble Angiopoietin-2 and ICAM-1 concentrations were dramatically elevated in patients with platelet count <10 × 10^9^/L and 10 × 10^9^ to 30 × 10^9^/L, compared with normal volunteers (**Table 2**). Moreover, the soluble ICAM-1 levels were significantly higher in patients with platelet count <10 × 10^9^/L than those with platelet count 30 × 10^9^ to 100 × 10^9^/L (**Table 2** **)**.

**Table 2:** Angiopoietin-2 and ICAM-1 concentrations in corticosteroid resistant ITP patients stratified by platelet count at enrollment.

Platelet count (10^9^/L)	<10 (n = 44)	10–29 (n = 26)	30–100 (n = 18)	Normal (n = 30)	*P*-value
**ICAM-1 (ng/ml),median (IQR)**	85 (63–103)^† ‡^	73 (55–107)^†^	84 (64–92)^†^	35 (24–48)	<0.05
**Angiopoietin-2 (pg/ml), median (IQR)**	1,346 (1,053–1,970)^†^	1,809 (1,200–2,755)^†^	1,501 (1,019–2,183)	606 (463–872)	<0.0001

^†^Significant vs. Normal (n = 20) using a Dunn’s test of multiple comparisons using rank sums.

^‡^Significant *vs*. Platelet count 30–100 (n = 10) using a Dunn’s test of multiple comparisons using rank sums.

IQR, interquartile range.

### ICAM-1 Might Predict Response to Low-Dose Decitabine Therapy

**Figure 3:**
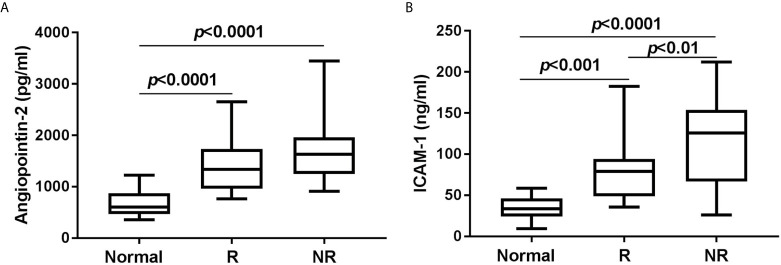
Plasma concentrations of Angiopoietin-2 **(A)** and ICAM-1 **(B)** at enrollment in healthy volunteers (Normal) and 29 corticosteroid resistant ITP patients who received consecutive low-dose decitabine therapy, including 15 responders (R) and 14 non-responders (NR).

In order to estimate the role of ICAM-1 and/or Angiopoietin-2 in predicting response to low-dose decitabine, we followed up 29 consecutive patients until achieving response or 90 days after the enrollment, whichever came first. Among these patients, 15 (52%) achieved response (nine complete responses and five partial responses) with platelet count ≥30 × 10^9^/L and at least two-fold increase than the baseline platelet count, without bleeding. However, another 14 patients (48%) presented no-response, without significant platelet increase or bleeding change. There was no difference in the frequency of glycoprotein autoantibodies against platelet GPIb/IX and/or GPIIb/IIIa complexes between the responsive group and the non-responsive group (data not shown). Compared with healthy volunteers, both responders and non-responders showed markedly higher Angiopoietin-2 and ICAM-1 concentrations at enrollment (**Figures 3A, B**). The difference of initial Angiopoietin-2 concentrations between responders and non-responders did not reach statistical significance. Notably, responders had significantly lower ICAM-1 concentrations, both before and after low-dose decitabine treatment, compared with non-responders [enrollment concentration 80(50, 92) pg/ml *vs*. 121(77–152) pg/ml, p < 0.01; end concentration 75(66,108) pg/ml *vs*. 123(100, 157) pg/ml, p < 0.05] (**Table 3**).

**Table 3:** Personal and laboratory characteristics of 29 corticosteroid resistant ITP patients who received consecutive low-dose decitabine therapy.

	Total (n = 29)	R (n = 15)	NR (n = 14)	P (R *vs*. NR)
**Age (years), median (range)**	43 (15–84)	39 (15–65)	49 (20–84)	0.275^a^
**Female, % (n)**	69 (20)	67 (10)	71 (10)	0.362^b^
**ITP duration (months), median (range)**	3 (1–12)	3 (1–12)	4 (1–12)	0.276^a^
**Previous therapies, % (n)**				
Corticosteroid****	100 (29)	100 (15)	100 (14)	–
rhTPO****	52 (15)	40 (6)	64 (9)	0.191^b^
** **Eltrombopag	17 (5)	13 (2)	21 (3)	0.564^b^
** **Intravenous Immunoglobulin	41 (12)	40 (6)	43 (6)	0.597^b^
**Platelet count (10^9^/L),** **median (range)**				
At enrollment****	7 (1–65)	6 (1–65)	6 (1–21)	0.442^a^
At end of observation****	35 (0–266)	119 (35–266)	7 (0–35)	<0.001^a^
**Bleeding score,** **median (range)**				
At enrollment****	3 (0–11)	3 (0–6)	3 (0–11)	0.946^a^
At end of observation****	0 (0–3)	0 (0–0)	2 (0–3)	<0.001^a^
**ICAM-1 (ng/ml),** **median (IQR)**				
At enrollment****	92 (59–121)	80 (50–92)	121 (77–152)	0.005^a^
At end of observation****	103 (70–136)	75 (66–108)	123 (100–157)	0.026^a^
**Angiopoietin-2 (pg/ml),** **median (IQR)**				
At enrollment****	1,559 (1,091–1,716)	1,291 (866–1,007)	1,633 (1262–1,837)	0.138^a^
At end of observation****	1,366 (1,027–1,778)	1,102 (999–1,436)	1,650(1167–1,852)	0.163^a^

a Mann–Whitney U test.

b Fisher exact or chi-square test.

R, responder; NR, non-responder; IQR, interquartile range.

Furthermore, logistic regression models were constructed to determine the association between clinical variables and lack of response to low-dose decitabine. The ICAM-1 level at enrollment was associated with no response through univariate logistic regression analysis (p < 0.05) and was included in the multivariable logistic regression model. For every 5 pg/ml increase in ICAM-1 concentration, the odds of no response were increased by 36.8% (OR 1.368, 95% CI 1.060–1.764) (**Table 4**).

**Table 4:** Logistic regression analysis evaluating predictors of no response to consecutive low-dose decitabine therapy for corticosteroid resistant ITP patients.

	OR	95% CIr		*P*-value
		Lower	Uppper	
**Univariable analysis of subject characteristics**				
Age	1.027	0.979	1.077	0.281
Male	2.000	0.446	8.963	0.365
Baseline platelet	0.947	0.860	1.043	0.272
Angiopoietin-2 (50 ng/ml increase)	1.084	0.991	1.186	0.078
ICAM-1 (5 pg/ml increase)	1.354	1.071	1.713	0.011
**Final multivariable prediction model**				
ICAM-1 (5 pg/ml increase)	1.368	1.060	1.764	0.016
Angiopoietin-2 (50 ng/ml increase)	1.082	0.947	1.235	0.247

Subject characteristics were chosen for the univariable analysis, and only two variables are shown in the final multivariable analysis. 95% CI indicates 95% confidence interval.

## Discussion

Vascular endothelial cells are versatile effectors involved in hemostasis, leukocyte recruitment, and movement of soluble and blood cellular elements (24, 25). Dana et al. found endothelial alterations in a canine model of immune thrombocytopenia (26). Endothelial cell activation/injury has been reported in ITP patients, with elevated levels of ICAM-1, thrombomodulin, and H3Cit-DNA (27). However, the pathogenetic role of endothelial dysfunction in corticosteroid resistant ITP remains unclear. To our best knowledge, this is the first study to demonstrate that plasma ICAM-1 significantly correlated with disease severity of corticosteroid resistant ITP patients, and initial ICAM-1 level might predict response to low-dose decitabine treatment.

Endothelial dysfunction is characterized by endothelial activation, pro-inflammatory and procoagulant changes, and damage of endothelial integrity. ICAM-1 is expressed by leucocytes and vascular endothelial cells and is considered as one marker of endothelial activation. In physiological condition, ICAM-1 keeps very low level in circulation, but its level may markedly elevate during endothelial dysfunction. Increased expression of ICAM-1 results in enhanced binding of leucocytes, which is attributable to local inflammatory response and endothelial cell damage (28). However the Angiopoietin/Tie2 signaling and VEGF axis are vital regulators of vascular integrity. It has been elucidated that these pro-angiogenic growth factors could not only accelerate angiogenesis but also regulate the immune homeostasis (29, 30). VEGF could limit maturation of DCs and the accumulation of myeloid immune cells (13). Angiopoietin-2, a ligand of TIE2/TEK receptor, could context-specifically act on the Tie2 receptor that activates the endothelium and reinforces permeability and vascular inflammation by altering adherence junctions, recruiting leukocytes and inducing disturbed thrombosis (31, 32).

In the current study, three soluble plasma biomarkers of endothelial activation (ICAM-1, Fractalkine, E-selectin) and three plasma biomarkers of vascular integrity (Angiopoietin-2, Angiopoietin-1, VEGF) were measured. Increased levels of ICAM-1, VEGF, and Angiopoietin-2 were found in corticosteroid resistant ITP patients compared with healthy controls, indicating the occurrence of endothelial dysfunction in ITP. This is consistent with previous report that endothelium activation is found before and after thrombopoietin receptor agonist treatment (27). Further analysis identified the association between the concentrations of these vital biomarkers and major clinical characteristics including platelet count and bleeding risk. Patients with severe thrombocytopenia exhibited higher levels of ICAM-1, demonstrating that enhanced endothelial activation and weakened vascular integrity are involved in disease aggravation. Previous research showed that Angiopoietin-2 treatment could stimulate ICAM-1 expression and significantly increase soluble ICAM-1 secretion from endothelial cells (33). Lang et al. reported that blockade of ICAM-1 with a neutralizing antibody could relieve Angiopoietin-2 induced macrophage recruitment and vascular dysfunction in hypertension (34). No association observed between ICAM-1 and Angiopoietin-2 in our study may be due to the small sample size. Previous reports have found that several pro-inflammatory cytokines could induce endothelial cells to release ICAM-1; the latter enhances activation of endothelium by disrupting endothelial tight junction and facilitating leukocyte transmigration (35). Chao et al. reported that TNF-α stimulation could markedly upregulate ICAM-1 through restraining NF-*κ*B activation and ROS signal in endothelial cells (36). Higher levels of TNF-α have been reported in the plasma/serum from ITP patients (37). Therefore, we propose that increased TNF-α in ITP patients may induce the release of ICAM-1, that in turn boosts endothelium activation, constructing a positive ongoing activation loop. We suppose that activated endothelium may attribute to persistence of chronic inflammation and activation of platelets, leading to increased platelet destruction indirectly.

We sought to exploit whether ICAM-1, the biomarker of endothelium dysfunction, could predict response to low-dose decitabine in ITP. Endothelial cells throughout the bone marrow have been dedicated to create niches for hematopoietic stem cells (38). Emerging evidence from murine studies suggest that bone marrow endothelial progenitor cells might regulate megakaryocytopoiesis and thrombopoiesis (39, 40). As we know, platelets are anucleated cell fragments derived from bone marrow megakaryocytes. The endothelial barrier could significantly regulate the mature megakaryocyte trafficking within the bone marrow that dedicate to new platelet production (41). Yuan et al. observed that corticosteroid resistant ITP patients showed reduced and dysfunctional bone marrow endothelial cells and atorvastatin could alleviate thrombocytopenia by improving bone marrow endothelial cell function in these patients (42). Our previous research demonstrated that low-dose decitabine promotes megakaryocyte maturation and platelet production in ITP (43). Recently the efficiency and safety of low-dose decitabine treatment have been verified in corticosteroid resistant ITP patients through a prospective multicenter study (10). In the current study, higher ICAM-1 could independently increase the negative risk in the achievement of response to low-dose decitabine, indicating that persistence of endothelial dysfunction might be attributable to refractory/resistance to existing ITP-specific treatments. Given the complex nature of ITP pathogenesis and the existence of close association between endothelium dysfunction and severity represented by platelet counts and bleeding risk in the present report, further studies are needed to clarify whether combination of targeting endothelium therapy and low-dose decitabine could exert synergistic effect on ITP.

This study had some limitations. The number of consecutive patients enrolled in the study was limited. Although the differences between patients stratified by respective characteristics were corrected through strict propensity matching, bias may have existed due to the unmeasured confounders. All the clinical data were collected from a single center in China, which might weaken the generalizability of findings. So large multi-center clinical trials with more patients are needed to validate the associations between endothelial dysfunction and ITP pathogenesis in the general population.

In summary, the clinical evidence suggested that corticosteroid resistant ITP patients exhibited ongoing endothelial dysfunction, especially those with low platelet count or severe bleeding. Moreover, our data demonstrate for the first time that the high plasma ICAM-1 level is correlated with non-response to low-dose decitabine treatment in corticosteroid resistant ITP. These data provide additional insight into the role of endothelial dysfunction in corticosteroid resistant ITP and a rational basis for testing therapeutics that repair impaired endothelium in corticosteroid resistant ITP patients.

## Data Availability Statement

The raw data supporting the conclusions of this article will be made available by the authors, without undue reservation.

## Ethics Statement

The studies involving human participants were reviewed and approved by Medical Ethical Committee of Qilu Hospital, Shandong University. The patients/participants provided their written informed consent to participate in this study.

## Author Contributions

LW and CL designed and performed research, analyzed data, and wrote the paper. LL, JS, and LS analyzed data and assisted the research. MH, JP, YH, and MX analyzed data and helped in funding this research. All authors reviewed the data, read, and edited the manuscript. All authors contributed to the article and approved the submitted version.

## Funding

This work was supported by grants from the National Natural Science Foundation of China (No.81100348, 81770114, 81900121, 81973994 and 81900124), Young Taishan Scholar Foundation of Shandong Province (No.tsqn201812133) and Natural Science Foundation of Shandong Province (ZR201807061123).

## Conflict of Interest

The authors declare that the research was conducted in the absence of any commercial or financial relationships that could be construed as a potential conflict of interest.
